# Methionine Residues in Exoproteins and Their Recycling by Methionine Sulfoxide Reductase AB Serve as an Antioxidant Strategy in *Bacillus cereus*

**DOI:** 10.3389/fmicb.2017.01342

**Published:** 2017-07-26

**Authors:** Jean-Paul Madeira, Béatrice M. Alpha-Bazin, Jean Armengaud, Catherine Duport

**Affiliations:** ^1^Sécurité et Qualité des Produits d'Origine Végétale (SQPOV), UMR0408, Avignon Université, Institut National de la Recherche Agronomique Avignon, France; ^2^Commissariat à lEnergie Atomique, Direction de la Recherche Fondamentale, Institut des Sciences du vivant Frédéric-Joliot (Joliot), Service de Pharmacologie et Immunoanalyse, Laboratoire Innovations Technologiques pour la Détection et le Diagnostic (Li2D) Bagnols-sur-Cèze, France

**Keywords:** methionine sulfoxide reductase, exoproteome, antioxidants, *Bacillus cereus*, metabolism

## Abstract

During aerobic respiratory growth, *Bacillus cereus* is exposed to continuously reactive oxidant, produced by partially reduced forms of molecular oxygen, known as reactive oxygen species (ROS). The sulfur-containing amino acid, methionine (Met), is particularly susceptible to ROS. The major oxidation products, methionine sulfoxides, can be readily repaired by methionine sulfoxide reductases, which reduce methionine sulfoxides [Met(O)] back to methionine. Here, we show that methionine sulfoxide reductase AB (MsrAB) regulates the Met(O) content of both the cellular proteome and exoproteome of *B. cereus* in a growth phase-dependent manner. Disruption of *msrAB* leads to metabolism changes resulting in enhanced export of Met(O) proteins at the late exponential growth phase and enhanced degradation of exoproteins. This suggests that *B. cereus* can modulate its capacity and specificity for protein export/secretion through the growth phase-dependent expression of *msrAB*. Our results also show that cytoplasmic MsrAB recycles Met residues in enterotoxins, which are major virulence factors in *B. cereus*.

## Introduction

Reactive oxygen species (ROS) are by-products of aerobic metabolism, and respiration is considered to be the major intracellular source of ROS production in bacteria (Brynildsen et al., 2013; Imlay, 2013). Methionine residues in proteins are particularly susceptible to oxidation by ROS (Vogt, 1995; Stadtman et al., 2005), resulting in methionine-*S*-sulfoxides [Met-S-(O)] and methionine-*R*-sulfoxides [Met-R-(O); (Luo and Levine, 2009; Kim et al., 2014)]. Oxidized methionine can be repaired by the antioxidant enzymes, Met-S-(O) reductase (MsrA) and Met-R-(O) reductase (MsrB). Both Msr share a common catalytic mechanism to reduce Met(O) back to Met. This catalytic mechanism leads to the formation of an intramolecular disulfide bond in the Msr and involves thioredoxin (Trx), thioredoxin reductase, and NADPH (Weissbach et al., 2002). It has been shown that MsrA reduces both oxidized proteins and low molecular weight Met(O)-containing compounds with a similar catalytic efficiency, whereas MsrB is specialized for the reduction of Met(O) in proteins. Interestingly, both Msr types preferentially repair unfolded proteins (Tarrago et al., 2012). The genes encoding MsrA and MsrB have been identified in most living organisms. Four different types of organization have been reported for *msrA* and *msrB*: (i) *msrA* and *msrB* genes are two separate transcription units, (ii) *msrA* and *msrB* cistrons are organized as an operon, (iii) *msr*A and *msrB* cistrons form a single open reading frame (ORF) to produce a two domain protein, and (iv) *trx, msrA*, and *msrB* cistrons form a single ORF to produce a three domain protein (Drazic and Winter, 2014).

Several studies have revealed the importance of Met oxidation and Msrs, especially regarding oxidative stress resistance and metabolism under stress conditions. In addition, Msrs have also been reported to be important virulence factors in pathogens by modulating a range of properties such as adherence (Wizemann et al., 1996; Giomarelli et al., 2006), motility (Hassouni et al., 1999), biofilm formation (Beloin et al., 2004), and *in vivo* survival (Alamuri and Maier, 2004). However, the importance of Met oxidation and Msr in the secretion of virulence factors under normal physiological conditions is largely unknown in pathogens, and in particular in *Bacillus cereus*.

*B. cereus* is a Gram-positive, motile human pathogen that is well-equipped to survive in various environments such as those encountered in soil, food and the human gastrointestinal tract (Stenfors Arnesen et al., 2008). These bacteria can grow in the presence or absence of oxygen (Rosenfeld et al., 2005; Duport et al., 2006). In the human intestine, *B. cereus* encounters oxic conditions in zones adjacent to the mucosal surface (Marteyn et al., 2010) and anoxic conditions in the intestinal lumen (Moriarty-Craige and Jones, 2004). In the presence of oxygen, *B. cereus* grows by means of aerobic respiration and secretes a large number of proteins into the extracellular compartment. These secreted proteins, and all the released proteins found in the pathogen's surrounding environment, constitute the *B. cereus* exoproteome (Clair et al., 2010, 2013; Laouami et al., 2014). We previously reported that the *B. cereus* exoproteome contained protein-bound Met(O) and that the accumulation of protein-bound Met(O) decreased significantly during aerobic respiratory growth, to reach its minimal value at the stationary phase (Madeira et al., 2015). Insofar as there is no ROS source and no Msr to reduce Met(O) back to Met in the extracellular milieu, we assumed that the time dynamic of protein-bound Met(O) in the *B. cereus* exoproteome could reflect the growth phase-dependent activity of an intracellular Msr. Here, we show that *B. cereus* encodes a functional MsrAB methionine sulfoxide reductase that is responsible for the decrease of the Met(O) content of the *B. cereus* exoproteome during aerobic respiratory growth. In addition, our results provide evidence that Met residues in exoproteins, especially enterotoxins, and their recycling by MsrAB, can serve as an antioxidant system that could trap ROS and maintain redox homeostasis in cells.

## Materials and methods

### Construction of a Δ*msrAB* mutant and its complementation

Mutant construction was performed according to the procedure developed by Arnaud et al. (2004). The *msrAB* ORF was interrupted by insertion of a non-polar spectinomycin resistance expression cassette, spc (Murphy, 1985) as follows. A DNA fragment of 1,413 bp encompassing the *msrAB* ORF was amplified from *B. cereus* genomic DNA by PCR with primers 5′-gaattcTCATGCCTTGAAAGTTACGG-3′ and 5′-agatctTTGGCGTAACGGTAATTGGT-3′, which contained *EcoR*I and *Bgl*II restriction sites, respectively. The amplified DNA fragment was cloned into pCRXL-TOPO (Invitrogen). The resulting pCRXL*msrAB* plasmid was digested with *Stu*I. A 1.5 kb *Sma*I fragment containing spc was purified from pDIA (Laouami et al., 2011) and ligated into *Stu*I-digested pCRXL*msrAB*. The resulting plasmid, pCRXL*msrAB*Δspc, was digested with *EcoR*I plus *Bgl*II. The *msrAB*Δspc fragment was then subcloned into *EcoR*I/*Bgl*II sites of pMAD (Arnaud et al., 2004). This construct was used for *B. cereus* transformation (Omer et al., 2015). For complementation of the Δ*msrAB* mutant with wild-type *msrAB* gene, the 1,413 bp *EcoR*I-*Bgl*II fragment was cloned into pHT304 (Arantes and Lereclus, 1991). *MsrAB* is under the control of its own promoter into pHT304-*msrAB*.

### *B. cereus* strains and growth conditions

Wild-type *B. cereus* ATCC 14579 without its pBClin15 plasmid (Madeira et al., 2016a,b), its Δ*msrAB* mutant and Δ*msrAB*/pHT304*msrAB* complemented strains were grown in MOD medium supplemented with 30 mM glucose as the carbon source, as previously described (Madeira et al., 2016b). The inoculum was a sample of exponential subculture harvested by centrifugation, washed and diluted in fresh medium to obtain an initial optical density at 600 nm of 0.02. Three independent batch cultures (biological replicates) were carried out at 37°C for each strain.

### Analytical procedures and growth parameters

*B. cereus* growth was monitored spectrophotometrically at 600 nm. The specific growth rate (μ) was determined using the modified Gompertz equation (Zwietering et al., 1990). Cells and filtered culture supernatants were harvested at the indicated growth stage as previously described (Madeira et al., 2015, 2016b). Exoproteins were immediately precipitated from the culture supernatant using trichloroacetic acid (TCA), as previously described, and stored at 4°C until analysis. The concentrations of substrate, and by-products in the filtered culture supernatants were determined with Enzytec Fluid kits purchased from R-Biofarm, as described by the manufacturer. Exoprotein concentration was determined by the Bradford protein assay (Pierce).

### Protein sample preparation, trypsin in-gel proteolysis, and nano-LC-MS/MS analysis

Protein extraction and subsequent digestion were performed as previously described (Madeira et al., 2015). Extracellular and intracellular proteins from the 27 samples (biological triplicates from the three time conditions for the wild-type, Δ*msrAB* and Δ*msrAB*/pHT304-*msrAB* strains) were resolved on NuPAGE® 4–12% Bis-Tris gels (Invitrogen) that were run for a short (about 3 mm) electrophoretic migration using NuPAGE MES supplemented with NPAGE antioxidant as the running buffer (Hartmann and Armengaud, 2014). This avoids artefactual protein oxidation. For each of the 54 protein samples, the whole protein content was extracted as a single polyacrylamide band. The bands were subjected to proteolysis with sequencing grade trypsin (Roche) following the ProteaseMAX protocol (Promega), as previously described (De Groot et al., 2009; Clair et al., 2010). NanoLC-MS/MS experiments were performed using an LTQ-Orbitrap XL hybrid mass spectrometer (ThermoFisher) coupled to an Ultimate 3000 nRSLC system (Dionex, ThermoFisher; Dedieu et al., 2011; Madeira et al., 2015).

### Peptide and protein identification from MS/MS datasets

MS/MS spectra were searched against an in-house polypeptide sequence database corresponding to an improved annotation of the *B. cereus* ATCC 14,579 genome (Madeira et al., 2016a). The MASCOT Daemon search engine (version 2.3.02; Matrix Science) was used to search tryptic peptides as previously described (Dupierris et al., 2009; Madeira et al., 2016a). The mass spectrometry proteomics data have been deposited in the ProteomeXchange Consortium (http://proteomecentral.proteomexchange.org) via the PRIDE partner repository (http://www.ebi.ac.uk/pride) with the dataset identifiers, PXD006169 and 10.6019/PXD006169 (exoproteome) and, PXD006205 and 10.6019/PXD006205 (cellular proteome).

### Label-free comparative proteomics

Analyses of changes of peptides and proteins in terms of abundance were achieved by comparing the spectral counts of proteins after voom transformation of abundance values using the R package LIMMA (Ritchie et al., 2015), as previously described (Madeira et al., 2016b). Data were normalized using the trimmed mean of *M*-values (TMM), implemented in the R package edgeR (Robinson et al., 2010). For quantitative comparisons, data were filtered to have two valid values in at least two biological replicates. Since we were specifically interested in the comparison between wild-type, Δ*msrAB* mutant and the complemented strain Δ*msrAB*/pHT-*msrAB*, we conducted differential analysis between WT and Δ*msrAB*, as well as Δ*msrAB* and Δ*msrAB*/pHT-*msrAB*, and WT and Δ*msrAB*/pHT-*msrAB*, individually. Differential protein and peptide abundances between WT and Δ*msrAB*, between Δ*msrAB* and Δ*msrAB*/pHT-*msrAB*, and between WT and Δ*msrAB*/pHT-*msrAB* were considered significant at stringent *p*-values (≤0.01). The results are presented as log_2_ fold-changes.

### Real-time RT-PCR and 5′RACE assays

Total RNA was prepared as described previously (Omer et al., 2015). Real-time RT-PCR was performed using the iScript™ One-Step RT-PCR kit with SYBR® Green following the manufacturer's protocol (Biorad). The *msrAB*-specific primer pair used in this study was: 5′-TTCTGGTACACAGGTGGTC-3′ and 5′-AAAGCGTCCACTCTGCTCAA-3′. Gene expression was normalized by the ΔΔCT analysis. The 16s rDNA was used as the reference gene in the calculations. The 16S rDNA-specific primer pair was 5′-TCCAACTGATGGCGGAC-3′ and 5′-TCACGCCCAGATTCTTTTTGC-3′. Rapid amplification of 5′ complementary cDNA ends (5′RACE) was performed using the 5′/3′ RACE kit (Sigma). The *msrAB* specific primers SP1, SP2 and SP3 were: 5′-ATGTCCCGTCGTTTCTGAAC-3, 5′-TCAAATGGCGAAACCATACA-3′ and 5′-CCATACACCAGAAGCACCCT-3′, repectively.

### Protease activity assay

Sigma's non-specific protease activity assay was used to determine the protease activity of filtered culture supernatant. In this assay, casein acts as a substrate. Tyrosine, which is released on hydrolysis of casein by proteases, is able to react with Folin-Ciocalteu's reagent to produce a blue chromophore. The quantity of this chromophore was measured by means of its absorbance value by spectrophotometry. Absorbance values generated by the activity of the protease were compared to a standard curve, which was generated on the basis of known quantities of tyrosine. From the standard curve, the activity of protease samples was determined in units, corresponding to the amount in micromoles of tyrosine equivalents released from casein per minute. Experiments were performed twice for each of the 27 filtered culture supernatants. Statistical differences were evaluated by the Student's *t*-test.

### Long-term survival

The survival of WT, Δ*msrAB* mutant, and complemented Δ*msrAB* mutant were determined as follows. After 24 h incubation at 37°C on glucose containing MOD medium, cultures were transferred to 4°C. An aliquot of each culture was collected before and after 1, 2, 3, 4, and 5 days of exposure to 4°C. Viable cells were determined by serial dilution of cultures in PBS, plating on LB agar, and incubation overnight (37°C). Experiments were performed in triplicate. Statistical differences were evaluated by the Student's *t*-test.

## Results

### *msrAB* expression is growth phase dependent

**Figure 1:**
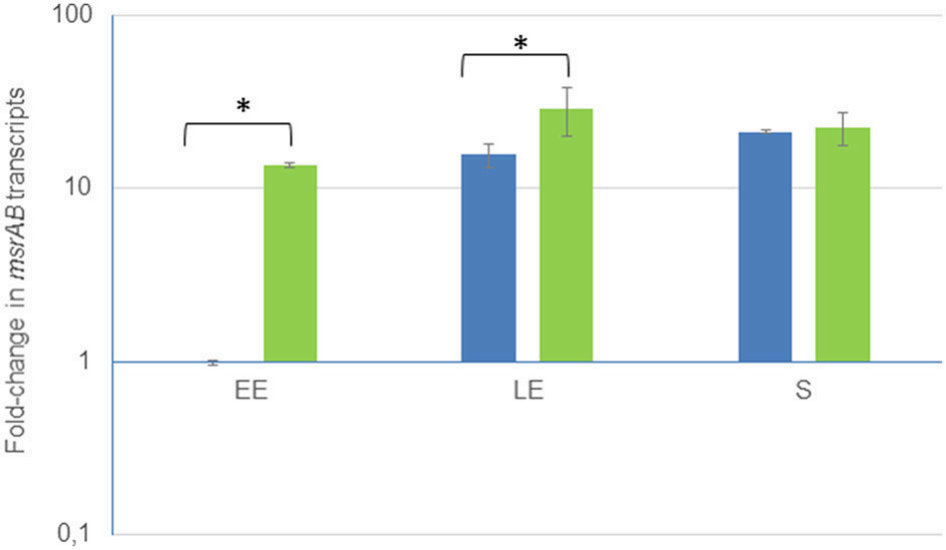
Growth phase-dependent changes of *msrAB* transcript levels in wild-type (blue) and complemented Δ*msrAB*/pHT304*msrAB* (green) strains. Fold changes refer to the levels observed in early exponential (EE) phase cultures of the WT strains. Significant differences are indicated with one (*p* < 0.05) asterisks. LE, Late exponential growth phase; S, Stationary growth phase.

Genome analyses of *B. cereus* ATCC 14579 identified an ORF (BC_5436) encoding a cytoplasmic protein annotated as MsrAB (NP_835097). This predicted cytoplasmic protein is composed of 321 amino acids and has a molecular weight of 36,938 Da. MsrAB and its gene *msrAB* are strongly conserved in members of the *B. cereus* group (data not shown). We mapped the transcriptional start site of *msrAB* by 5′RACE. The transcriptional start site (G) was located 23 nt upstream of the translational start codon and was preceded by a region similar to σE consensus-35 (TAATATG) and -10 (CATACTG) boxes separated by 13 nt. Furthermore, *msrAB* appeared to be followed by an inverted repeat (ΔG° = 23.6 kcal/mol) that may a transcriptional terminator (Figure S1). This indicates that *msrAB* may be transcribed as a single unit. To determine whether there is any regulation of *msrAB*, mRNA levels were measured at early exponential (EE), late exponential (LE) and stationary (S) growth phases. Figure 1 shows that there was about a 30-fold increase in *msrAB* expression for cells harvested at the S growth phase compared with the EE growth phase. *B. cereus msrAB* expression was thus maximal over the stationary phase. Similar stationary phase-induced expression of *msr* genes has been documented in several bacteria (Moskovitz et al., 1995; Vattanaviboon et al., 2005; Alamuri and Maier, 2006; Singh and Singh, 2012).

### MsrAB contributes to *B. cereus* respiratory metabolism

To investigate the role of MsrAB in *B. cereus*, we constructed a non-polar Δ*msrAB* mutant and a Δ*msrAB*-complemented strain using a multicopy pHT304-based plasmid (Arantes and Lereclus, 1991). Expression of *msrAB* in the complemented strain was under the control of its own promoter. We did not detect *msrAB* mRNA by RT-PCR in the mutant, proving that the genomic disruption of the gene generated an *msrAB*-null mutant. Figure 1 shows that *msrAB* was overexpressed in the strain Δ*msrAB*/pHT304*msrAB* at the EE and LE growth phases. Therefore, *msrAB* expression level was not restored by complementation.

**Figure 2:**
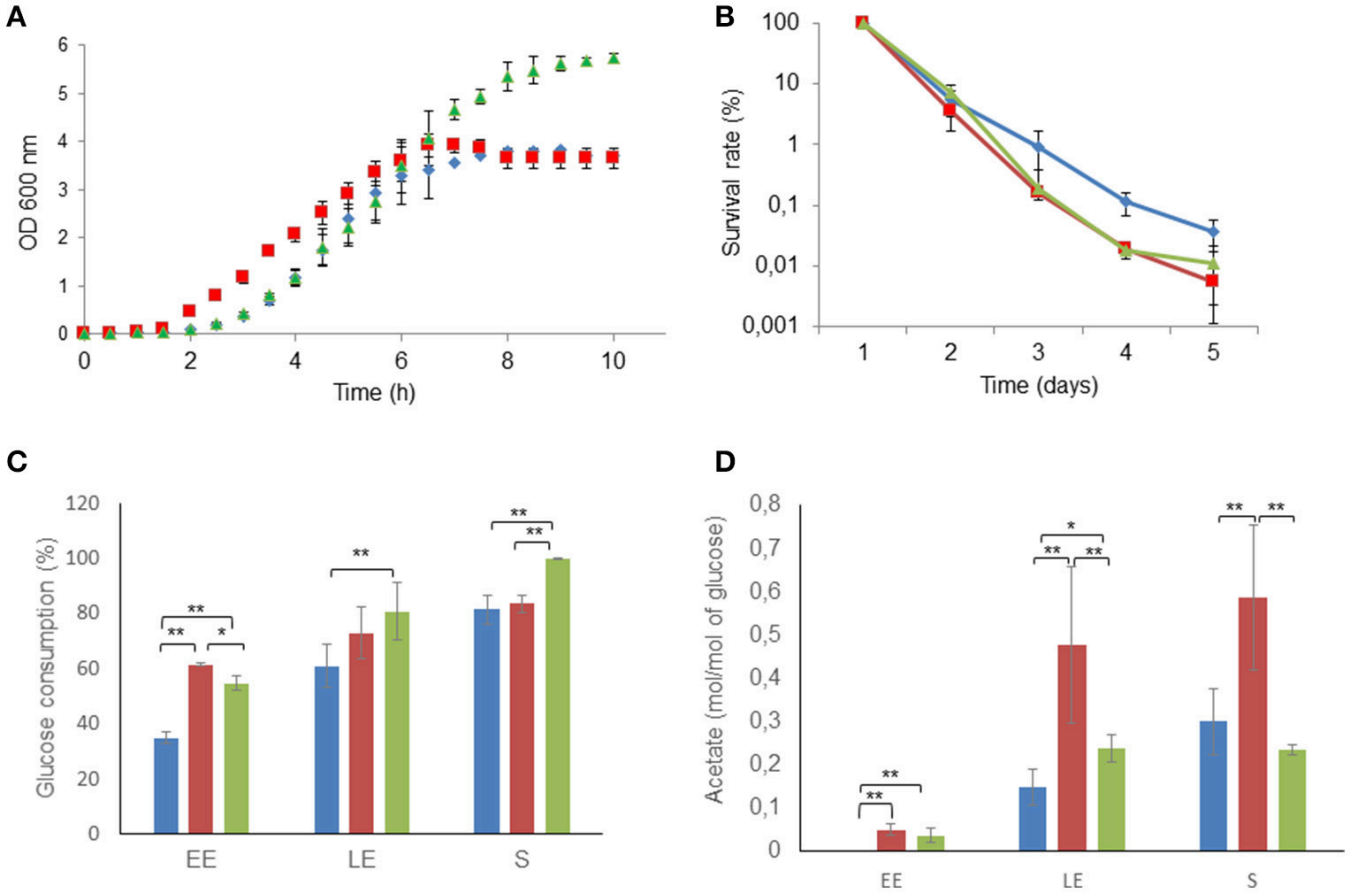
Altered growth and long-term survival of Δ*msrAB* mutant cells and complemented Δ*msrAB*/pHT304*msrAB* cells. **(A)** Growth curves of WT (blue), Δ*msrAB* (red) and Δ*msrAB*/pHT304*msrAB* (green) cells in pH-regulated batch cultures under aerobiosis. **(B)** Long-term survival of WT (blue), Δ*msrAB* (red) and Δ*msrAB*/pHT304*msrAB* (green) cells after growth under aerobiosis. **(C)** Glucose consumption of WT (blue), Δ*msrAB* (red) and Δ*msrAB*/pHT304*msrAB* (green) cells. **(D)** Acetate production of WT (blue), Δ*msrAB* (red) and Δ*msrAB*/pHT304*msrAB* (green) cells. Significant differences are indicated with one (*p* < 0.05) or two (*p* < 0.01) asterisks.

The growth characteristics of the three strains, Δ*msrAB*, Δ*msrAB*/pHT304*msrAB*, and the parental wild-type strain (WT), were determined under pH-regulated aerobic respiratory conditions in synthetic MOD medium. Figure 2A shows that the lag phase was 2.5-fold lower in the Δ*msrAB* strain (0.7 ± 0.1 h^−1^) than in the Δ*msrAB*/pHT304*msrAB* (1.8 ± 0.9 h^−1^) and WT (1.9 ± 0.2 h^−1^) strains. Exponential growth kinetics were similar in the three strains for the first 6 h. After this initial growth time, WT and Δ*msrAB* cultures entered stationary phase. In contrast, Δ*msrAB*/pHT304*msrAB* continued to grow and reached the stationary growth phase at a higher final biomass (2.6 ± 0.1 g.L^−1^) than Δ*msrAB* (1.9 ± 0.1 g.L^−1^) and WT (1.8 ± 0.2 g.L^−1^). The viabilities of Δ*msrAB* and Δ*msrAB*/pHT304*msrAB* cells, harvested at S growth phase, were similar to the viability of WT after 2 days but declined by more than 100-fold after 5 days of storage at 4°C (Figure 2B). This suggests that *msrAB* expression impacts the metabolic activity of *B. cereus* cells at the end of growth (Chubukov and Sauer, 2014). Figure 2C shows that the Δ*msrAB* and Δ*msrAB*/pHT304*msrAB* strains consumed higher amounts of glucose than WT at the beginning of exponential growth. The Δ*msrAB*/pHT304*msrAB* culture could be distinguished from the Δ*msrAB* culture by continued glucose consumption between the LE and S growth phases (Figure 2C). At the end of growth, Δ*msrAB*/pHT304*msrAB* consumed a higher level of glucose than Δ*msrAB* and WT. During aerobic respiratory growth, glucose is catabolized into CO_2_ through the TCA cycle, and acetate is excreted as a by-product of overflow metabolism (Madeira et al., 2015; Duport et al., 2016). Figure 2D shows that Δ*msrAB* cells, and to a lesser extent Δ*msrAB*/pHT304*msrAB* cells, excreted higher amounts of acetate than WT cells during exponential growth. Acetate accumulation stopped at the LE growth phase in the Δ*msrAB* and Δ*msrAB*/pHT304*msrAB* cultures while it continued to accumulate between the LE and S growth phases in the WT culture. Taken together, these results suggest that *msrAB* expression impacts on the metabolic activity of *B. cereus* under aerobiosis.

**Figure 3:**
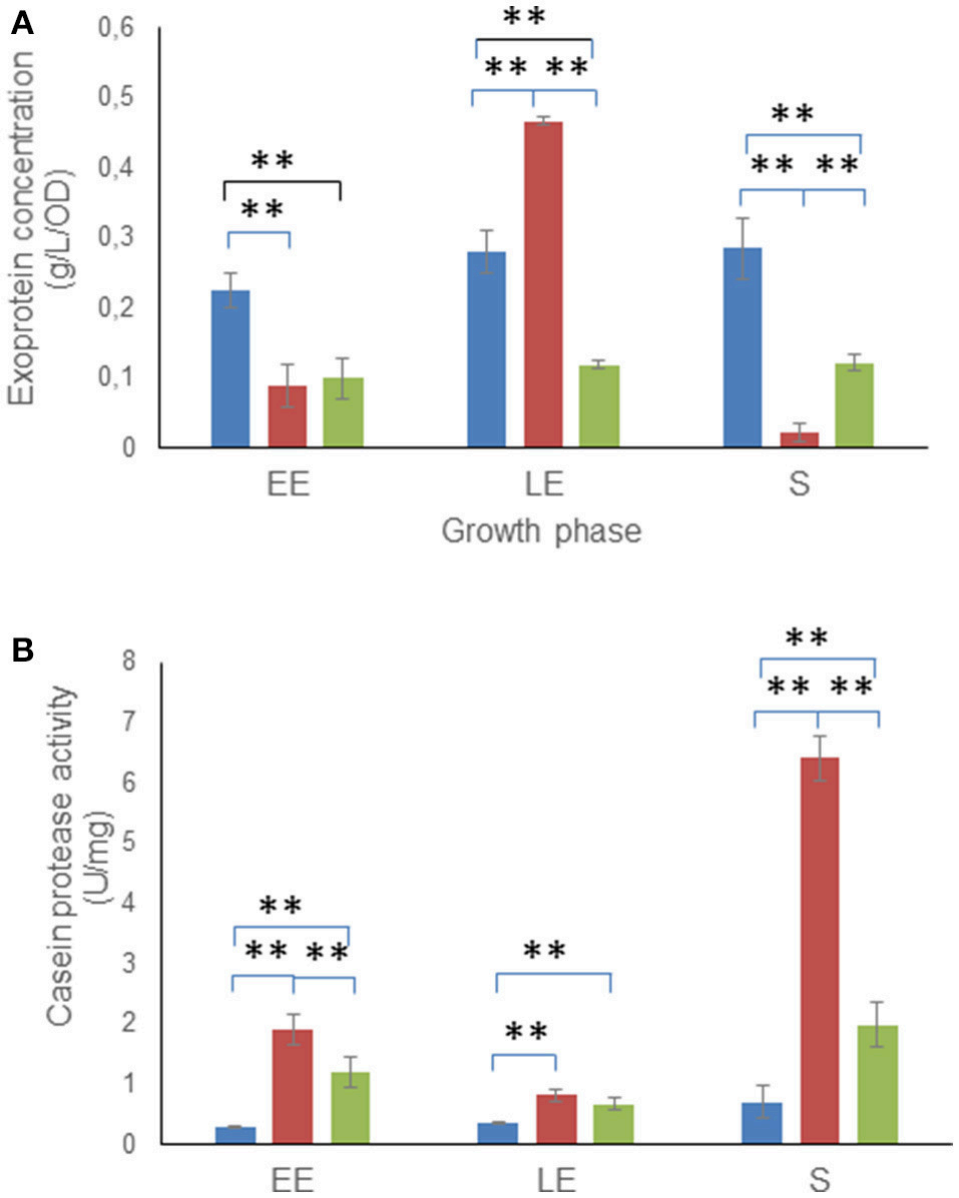
Exoproteome concentration and protease assay. Concentrations **(A)** and casein proteolytic activity **(B)** are indicated for total extracellular proteins of WT (blue), Δ*msrAB* (red) and Δ*msrAB*/pHT304*msrAB* (green) cells. Error bars represent the standard deviation from two independent measures for each biological triplicate. Significant differences (*p* < 0.01) between WT, Δ*msrAB* and Δ*msrAB*/pHT304*msrAB* strains are indicated with two asterisks.

To determine whether the alteration of glucose catabolism was associated with changes in extracellular protein production, extracellular proteins were extracted from culture supernatants of the three *B. cereus* strains, harvested during the EE, LE, and S growth phases (Madeira et al., 2015). Figure 3A shows that the Δ*msrAB* culture supernatant accumulated a higher amount of exoproteins than that of WT at the LE phase. However, Δ*msrAB* supernatant had 50 and 90% fewer exoproteins in the EE and S growth phases, respectively, compared with WT. This decreased exoprotein concentration could have resulted from a higher protease activity in the Δ*msrAB* culture supernatant. To test this hypothesis, we quantified the protease activity of the Δ*msrAB*, Δ*msrAB*/pHT304*msrAB* and WT culture supernatants against casein. Figure 3B shows that the Δ*msrAB* culture supernatant sustained a higher protease activity than WT, markedly in the EE and S growth phases. These changes in protease activity were only partially rescued in Δ*msrAB*/pHT304*msrAB*. However, unlike Δ*msrAB*/pHT304*msrAB*, there was no correlation between the protease activity and the amount of exoproteins in Δ*msrAB* at LE phase (Figure 3A). This indicates that changes in *msrAB* expression could be selective for certain extracellular proteases.

### MsrAB modulates the proteome profile of *B. cereus*

**Figure 4:**
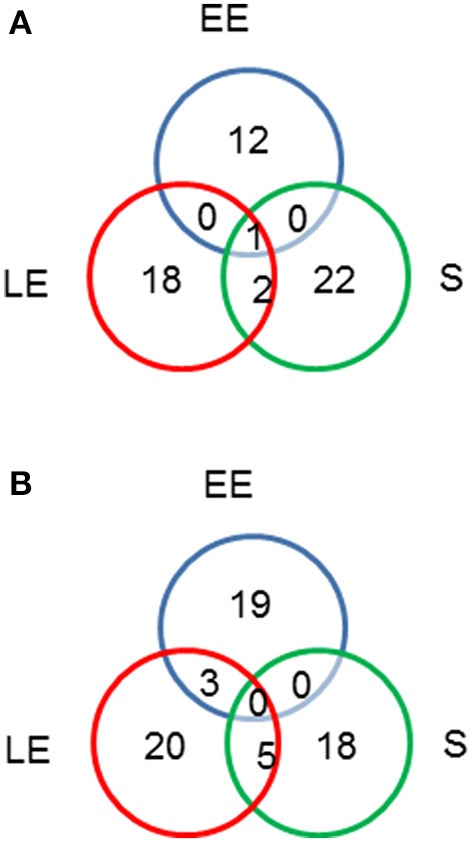
Distribution of proteins showing abundance level changes (*p* < 0.05) between wild-type and Δ*msrAB* mutant strains. Venn diagrams showing the number of regulated intracellular proteins **(A)** and exoproteins **(B)** in the Δ*msrAB* mutant in the early exponential (EE), late exponential (LE) and stationary (S) growth phases.

To determine if altered metabolism in Δ*msrAB* and Δ*msrAB*/pHT304*msrAB* was associated with cellular and exoproteome profile changes, we quantified protein abundance level differences between Δ*msrAB*, Δ*msrAB*/pHT304*msrAB*, and WT cells in the EE, LE and S growth phases. Exoproteome and cellular proteome samples were prepared from supernatant cultures and whole-cell lysates, respectively. A total of 200,746 and 71,676 MS/MS spectra were recorded from cellular proteome and exoproteome samples, respectively. A total of 922 proteins were identified in the cellular proteome (Table S1) and 371 proteins were identified in the exoproteome (Table S2), based on the confident detection of at least two different peptides. A two-sample *t*-test was then conducted separately between WT and Δ*msrAB*, and between Δ*msrAB* and Δ*msrAB*/pHT304*msrAB*. All proteins with a *p* ≤ 0.01 and at least a 2-fold change (log_2_ fold-change ≥ 1) were considered to be differentially modulated in terms of abundance. A total of 64 and 78 proteins were found to vary in abundance in Δ*msrAB* compared with WT in the cellular proteome and exoproteome fractions, respectively. The majority (80%) of these proteins were not rescued in Δ*msrAB*/pHT304*msrAB* (data not shown). The Venn diagrams presented in Figure 4 show the growth phase distribution of the identified proteins. Less than 2% of proteins showed abundance level changes in all three growth stages, indicating that *msrAB* modulates *B. cereus* cellular and exoproteome mainly in a growth phase-dependent manner. The impact of *msrAB* disruption appeared to be more important at the LE and S than the EE growth phase in the cellular proteome (Figure 4A), according to its expression (Figure 1).

#### Cellular proteins

Table 1 lists the identities and putative functions of the cellular proteins differentially produced in Δ*msrAB* compared with WT. In the EE phase, three proteins impacted by *msrAB* disruption were classified as carbohydrate metabolism enzymes. The glycolytic enzyme, Tpi (triose phosphate isomerase), and the two TCA enzymes, Mqo (malate:quinone oxidoreductase) and FumB (fumarate hydratase), were less abundant in Δ*msrAB* than WT. Tpi catalyzes the interconversion of dihydroacetone phosphate (DHAP) and glyceraldehyde-3-phosphate to prevent DHAP accumulation. It has been shown that a reduction of Tpi activity redirected the carbon flux from glycolysis to the pentose phosphate pathway (PPP), which provides the redox power for antioxidant systems (Ralser et al., 2007). FumB catalyzes the reversible hydration of fumarate to malate, and Mqo oxidizes malate to oxaloacetate and reduces quinone via a one-transfer electron reaction (Kabashima et al., 2013). By decreasing FumB and Mqo levels, Δ*msrAB* cells can thus decrease TCA activity, and consequently respiratory chain activity and ROS production. The reduction of respiratory chain capacity could be compensated by increasing glycolytic flux and increasing overflow metabolism (acetate excretion), as observed in Figure 2. Only one stress response-related protein (USP) was shown to be differentially produced in Δ*msrAB* at EE phase. As recently reported, USP may function as a protein regulator of downstream effectors of nucleotide-binding protein cycling. This activity depends on the energy status (ATP level) of the cells (Banerjee et al., 2015). A decrease in the UPS abundance level in Δ*msrAB* could thus reflect a change in ATP availability and/or demand at EE phase. Δ*msrAB* also sustained a higher level of RibD whatever the growth phase. The gene encoding RibD belongs to the putative operon *ribDEAH*, which encodes RibD, a pyrimidine deaminase/reductase, RibE, the α-subunit of riboflavin synthase, RibA, the GTP cyclohydrolase/3,4-dihydroxy 2-butanone 4-phosphate (3,4-DHBP) synthase, and RibH, the β-subunit of riboflavin (RibH). These enzymes form a pathway that produces one riboflavin molecule from GTP and ribulose-5-phosphate (Vitreschak et al., 2002). RibA and RibH were more highly produced in Δ*msrAB* than in WT at LE growth phase and RibE was more highly produced at S growth phase. Together these results suggest the increased production in Δ*msrAB* of riboflavin, which is known to be an element of antioxidant defense (Abbas and Sibirny, 2011). One stress-related protein, named AcpD (annotated as an azoreductase), which was not detected in WT cells (Table S1), was significantly induced at both LE and S growth phases in Δ*msrAB* cells. AcpD is a putative FMN-NAD(P)H-dependent quinone oxidoreductase that catalyzes the two-electron reduction of quinones to quinols. This protein could play an important role in managing oxidative stress in the absence of *msrAB* by maintaining the reduced antioxidant form of quinone (Ross et al., 2000; Ryan et al., 2014). Several proteins related to the biosynthesis of amino acids were upregulated at the LE and S phases. This suggests that an increase in the intracellular content of these amino acids may be part of the adaptive response to the lack of MsrAB.

**Table 1:** Cellular proteins with significant abundance level changes (|log_2_|fold-change > 1, *p* < 0.01) in Δ*msrAB* compared with WT.

**Functional class**	**NP no**.	**Gene no**.	**Protein name**	**Protein description**	**Log_2_ fold-change**		
					**EE**	**LE**	**S**
Carbohydrate metabolism	**NA**	**BC5137**	**Tpi**	**Triosephosphate isomerase**	−1.50		
	NP_834982	BC5320	Ccr	PTS system, glucose-specific IIA component		1.82	
	NP_834343	BC4637	Ack	acetate kinase			1.09
	NP_832706	BC2959	Mqo	Malate:quinone oxidoreductase	−2.82		
	NP_831487	BC1712	FumB	Fumarate hydratase	−2.57		
	NP_833692	BC3973	PdhA	Pyruvate dehydrogenase E1 component alpha subunit			1.02
	NP_833555	BC3834	SucC	Succinyl-CoA synthetase subunit beta			1.05
Enterotoxin	NP_834610	BC5239	EntA	Enterotoxin A			−4.07
Lipid metabolism	**NP_830401**	**BC0584**		**Acetyltransferase**			−2.04
Cell wall and cell surface metabolism	NP_830495	BC0682	SrtA	Sortase		−3.01	
	NP_834255	BC4548	IsdA1	Cell surface protein			−4.19
Purine metabolism	NP_832069	BC2306	BacF	Glycine-AMP ligase		−3.94	
	**NP_831124**	**BC1343**	**QueE**	**Organic radical activating protein**			2.63
	**NP_831122**	**BC1341**	**QueC**	**Aluminum resistance protein**			3.28
Pyrimidine metabolism	NP_833606	BC3886	CarB	Carbamoyl phosphate synthase large subunit		−4.35	
	NP_833803	BC4085	Pdp	Pyrimidine-nucleoside phosphorylase		2.14	
DNA binding and repair	NP_831634	BC1861		Helicase	−3.15		
	NP_831628	BC1855		Chromosome segregation ATPase	−2.46		
	NP_834171	BC4459	HsdM	Type I restriction-modification system methylation subunit		−2.51	
	NP_831628	BC3769	MutS	DNA mismatch repair protein		−2.58	
Aminoacid metabolism	NP_833492	BC1546	Aat	Aspartate aminotransferase			1.74
	NP_831735	BC1965	ThrC	Threonine synthase		3.41	3.13
	NP_831736	BC1966	ThrB	Homoserine kinase		2.64	3.30
	NP_832070	BC2307		Glycine-AMP ligase		−3.31	
	NP_831552	BC1779	IlvC2	Ketol-acid reductoisomerase			1.58
	**NP_831190**	**BC1410**	**HisF**	**Imidazole glycerol phosphate synthase subunit HisF**		2.05	
	**NP_831186**	**BC1406**	**HisD**	**Histidinol dehydrogenase**			3.41
	NP_831734	BC1964	Hom1	Homoserine dehydrogenase		2.86	2.35
	**NP_830438**	**BC4331**	**AroE**	**Shikimate 5-dehydrogenase**	2.06		
Amino sugar metabolism	NP_834865	BC5201	MnaA	UDP-N-acetylglucosamine 2-epimerase			1.59
Translation	**NP_831277**	**BC1498**	**RrpsA**	**30S ribosomal protein S1**	−3.21		
	**NP_830015**	**BC0135**	**RpsS**	**SSU ribosomal protein**	2.06		
Motility	NP_831407	BC1629	CheC	Flagellar motor switch protein		−2.38	
	NP_831428	BC1651	FglE	Flagellar hook protein			−3.26
	NP_831435	BC1658	FlaB	Flagellin		−2.30	
	NP_831415	BC1637	FlgL	Flagellar hook-associated protein			−3.28
	NP_834158	BC4446	MreB	Rod shape-determining protein			1.05
Rod shape-determining proteins	NP_834531	BC4831		ABC transporter ATP-binding protein		3.67	
Transporters	NP_834524	BC4824		ABC transporter ATP-binding protein		−2.11	
	NP_833512	BC3790		Nucleoside transport ATP-binding protein		−3.36	
	NP_830967	BC1182	OppD	Oligopeptide transport ATP-binding protein		−1.78	
	NP_832817	BC3071	CutC	copper homeostasis protein cutC			3.00
	**NP_834331**	**BC4625**	**UspA**	**Universal stress protein**	−3.19		
Stress response	**NP_835071**	**BC5410**	**AcpD**	**Azoreductase**		4.87	5.83
	NP_830954	BC1168	ClpB	ATP-dependent chaperone	−2.99		
Chaperones	NP_830829	BC1043	PrsA1	Peptidylprolyl isomerase			1.08
	NP_833827	BC4109	RibD	Diaminohydroxyphosphoribosylaminopyrimidine deaminase	2.78	2.92	4.42
Riboflavin biosynthesis	NP_833829	BC4111	RibA	Bifunctional 3,4-dihydroxy-2-butanone 4-phosphate synthase		2.87	
	**NP_833828**	**BC4110**	**RibE**	**Riboflavin synthase subunit alpha**			2.20
	NP_833830	BC4112	RibH	Riboflavin synthase subunit beta		1.68	
	NP_833832	BC4114	BioB	Biotin synthase			3.40
Biotin biosynthess	**NP_831123**	**BC1342**		**6-pyruvoyl tetrahydrobiopterin synthase**	2.16		
Folate biosynthesis	NP_833540	BC3819	Dxr2	1-deoxy-D-xylulose 5-phosphate reductoisomerase	2.75		
Terpenoid backbone biosynthesis	NP_831099	BC1317	PhaB	Acetoacetyl-CoA reductase	−2.53		
Uncategorized	NP_829927	BC0025		Unknown		2.67	
	NP_832675	BC2927		Prolyl endopeptidase			4.18
	NP_831667	BC1894		Phage protein	−2.84		
	NP_831673	BC1901		phage protein			−2.43
	NP_834610	BC4938		NADH dehydrogenase		2.03	
	NP_834043	BC0622		L-threonine 3-dehydrogenase			2.14
	NP_830802	BC1016		Unknown		3.04	
	NP_834559	BC4860		Unknown			−1.97
	NP_829986	BC0105		Unknown			1.87
	NP_834083	BC4371		Unknown			1.57

*Proteins showing abundance level restored in ΔmsrAB/pHT304msrAB are indicated in bold. EE, early exponential growth phase; LE, late exponential growth phase; S, stationary growth phase. NA, Not Annotated. Green and red highlights indicate increased and decreased protein levels, respectively*.

A protein was considered validated when at least two different peptides were found in the same sample. We found only one peptide assigned to MsrAB and did not validate its presence in the cellular proteome. To determine whether MsrAB is a true cellular protein, we carried out further analyses using a Q-exactive HF mass spectrometer. Five and 19 peptides assigned to MsrAB were detected in the cellular proteome of WT and Δ*msrAB*/pHT304*msrAB*, respectively, at LE and S growth phases (Figure S1) No peptide was detected in the exoproteome, proving that MsrAB is cytoplasmic.

#### Exoproteome

Table 2 lists the exoproteins that were considered as differentially produced in Δ*msrAB* supernatant. The majority of the metabolism and stress/detoxification-related proteins were less abundant in Δ*msrAB* compared with WT, regardless of growth phase. These proteins were predicted to be cytosolic and, accordingly, we found that they were more abundant in the cellular proteome compared with the exoproteome (Table S3). In contrast, the majority of the cell wall/surface-associated proteins, transporters and degradative/adhesin proteins, which were predicted to be secreted proteins, were increased in Δ*msrAB* compared with WT, especially at the EE and LE growth phases. This suggests that *msrAB* deletion could favor the accumulation of some secreted exoproteins at the expense of cytosolic proteins. Interestingly, two predicted secreted foldases, PrsA1 and PsrA2, showed significant increases in their abundance levels in Δ*msrAB*, especially at LE growth phase. PrsA1 and PrsA2 have been predicted to function as peptidyl-prolyl isomerases at the bacterial membrane–cell wall interface, to assist in the folding and stability of exported proteins (Vitikainen et al., 2004). In addition, we noted increased abundance levels of a bacterial type I signal peptidase protein (SPase) in Δ*msrAB* compared with WT at LE phase. SPases function at the terminal step of the general secretory pathway by releasing translocated proteins from the cytoplasmic membrane at a defined cleavage site (Craney et al., 2015). This Spase could thus function in conjunction with PrsA proteins to sustain a higher secretion level of some proteins (Alonzo et al., 2011).

**Table 2:** Exoproteins with significant abundance level changes (|log2|fold-change > 1, *p* < 0.01) in Δ*msrAB* compared with WT.

**Functional class**	**NP no**.	**Gene no**.	**Protein name**	**Protein description**	**Log_2_ fold-change**		
					**EE**	**LE**	**S**
Metabolism	NP_833767	BC4049	HPr	Phosphocarrier protein HPr			−2.24
Carbohydrate	NP_834306	BC4600	Pfk	6-phosphofructokinase	−3.09		
	NP_833689	BC3970	PdhD	Dihydrolipoamide dehydrogenase		−2.43	
	**NA**	**BC5138**	**Pgk**	**Phosphoglycerate kinase**		−1.79	
	NP_834571	BC4898	Pgi	Glucose-6-phosphate isomerase		−2.30	
	NP_833346	BC3616	Acn	Aconitate hydratase	−3.30		
Fatty acid and phospholipid	NP_833485	BC3761	PlcA	1-phosphatidylinositol phosphodiesterase precursor		1.15	
Amino acids	NP_830183	BC0344	RocA	1-pyrroline-5-carboxylate dehydrogenase	−3.18		
	**NA**	**BC3705**	**GlnA1**	**Glutamine synthetase, type I**		−3.45	
	NP_834978	BC5316	GlyA	Serine hydroxymethyltransferase	−2.58		
	NP_831022	BC1238	TrpA	Tryptophan synthase subunit alpha	−2.21	−2.67	
	**NP_833521**	**BC3799**	**Asd**	**Aspartate-semialdehyde dehydrogenase**		−1.53	2.30
	NP_834652	BC4981	DcyD	Cysteine desulfhydrase	−1.87		
	**NP_830053**	**BC0185**	**RocF**	**Arginase**		−1.57	
Amino sugar and nucleotide sugar	NP_830056	BC0188	GlmM	Phosphoglucosamine mutase			3.81
Nucleotide	NP_835123	BC5468	AdSS	Adenylosuccinate synthetase	−2.53		
Butanoate	NP_831099	BC1317	PhaB	Acetoacetyl-CoA reductase	−2.56		
Gamma Hexachlorocyclohexane degradation	NP_834220	BC4511	LppC	Acid phosphatase			−2.34
Ubiquinone and other terpenoid-quinone	NP_832068	BC2305	DhbB	Isochorismatase	2.45		
Toxins	NP_832699	BC2952	EntB	Enterotoxin/cell-wall binding protein	2.25		
	NP_832844	BC3101	HblB	Hemolysin BL binding component precursor		1.69	
	NP_833256	BC3523	HlyII	Hemolysin II		−1.78	
Degradative enzymes & adhesins	NP_83404223	BC4514	VanY4	D-alanyl-D-alanine carboxypeptidase	1.77		
	NP_833486	BC3762	Sfp	subtilisine like serine protease	3.14		
	**NP_830673**	**BC0887**	**CnaA**	**Collagen adhesion protein**	2.96		3.19
	NP_831437	BC1660	MltB	Soluble lytic murein transglycosylase	1.65		
	NP_835018	BC5357	CnaC	Collagen adhesion protein	1.77		
	NP_835020	BC5359	YwaD	Aminopeptidase Y		2.13	
	**NP_830419**	**BC0602**	**Npr600**	**Bacillolysin**		2.70	
	NP_832233	BC2473	Blm	Beta-lactamase		2.05	
	NP_831066	BC1284	InhA2	Immune inhibitor A precursor			−4.17
	NP_831063	BC1281	CalY	Cell envelope-bound metalloprotease (camelysin)			−2.85
	NP_830483	BC0670	PlcB	Phospholipase C			−2.42
Motility	NP_831428	BC1651	FglE	Flagellar hook protein	2.63		
	**NP_831414**	**BC1636**	**FlgK**	**Flagellar hook-associated protein**	2.66	−3.63	
	NP_831435	BC1658	FlaB	Flagellin		−3.70	
	NP_831415	BC1637	FlgL	Flagellar hook-associated protein			−4.14
	NP_831421	BC1643	FliE	Flagellar hook-basal body protein			−2.72
Cell wall and cell surface associated proteins	NA	BC3763		Cell wall hydrolase	3.31		
	NP_831197	BC1417	YvgJ3	phosphoglycerol transferase	2.61		
	NP_831682	BC1911	Ami	N-acetylmuramoyl-L-alanine amidase	−2.73		
	NP_830492	BC0679	Smc	Cell wall-binding protein	1.12		
	NP_832677	BC2929	PgdA	Peptidoglycan N-acetylglucosamine deacetylase		2.67	
	NP_831846	BC2078		Hypothetical Membrane Spanning Protein			−3.07
	NP_832595	BC2846	DltD	Protein dltD precursor			−2.25
	NP_833426	BC3698	CwpC	Cell wall endopeptidase			2.34
	NP_833984	BC4270		Penicillin-binding protein	3.48	1.94	
	NP_833266	BC3533	VanW	Vancomycin B-type resistance protein vanW	1.67		
Protein export	NP_832816	BC3070	SipA	Signal peptidase I		2.04	
Transport	NP_831789	BC2021	ZnuA	High-affinity zinc uptake system protein	1.48		
	NP_834656	BC4985		ABC transporter substrate-binding protein			−3.93
	NP_830083	BC0215	OppA2	Oligopeptide-binding protein oppA			−2.53
	NP_830606	BC0816		Periplasmic component of efflux system		3.09	2.50
	NA	BC1596		Permease	3.60	2.54	
Stress/detoxification	NP_831779	BC2011	Dps1	Non-specific DNA-binding protein	−2.31	−2.32	
	**NP_834714**	**BC5044**	**Dps2**	**Non-specific DNA-binding protein**		−3.47	
	**NP_833272**	**BC3539**	**CspB**	**Cold shock protein**		−5.13	−3.92
	**NP_830215**	**BC0376**	**AhpF**	**Alkyl hydroperoxide reductase subunit F**		−1.99	
Protein folding	NP_830947	BC1161	PrsA2	Peptidylprolyl isomerase		2.80	
	NP_830829	BC1043	PrsA1	Peptidylprolyl isomerase		2.57	
	**NP_834192**	**BC4480**	**Tig**	**Trigger factor**			−2.14
Translation	NP_830029	BC0149	RpmD	50S ribosomal protein L30	1.30		
	NP_833528	BC3806	RpsO	30S ribosomal protein S15			−2.38
	NP_830008	BC0128	FusA	Elongation factor G		−2.59	
	NP_830009	BC0129	Tuf	Elongation factor Tu		−2.79	2.95
Transcriptional regulators	NP_830591	BC0801	LytR2	LytR family transcriptional regulator	1.98		
	NP_834928	BC5265	LytR1	LytR family transcriptional regulator	2.88		
	NP_831739	BC1669	LytR3	LytR family transcriptional regulator		3.42	2.73
Cell division	NP_829962	BC0065		Cell division protein DIVIC		3.39	
Uncategorized	NP_831643	BC1870		Phage protein	1.30		
	NP_831665	BC1892		Phage protein		−1.58	
	NP_831667	BC1894		Phage protein		−1.89	−1.70
	NP_831675	BC1903		Phage protein			−2.16
	NP_832991	BC3251		Unknown		−2.71	
	NP_835021	BC5360		Unknown		2.05	
	NP_830068	BC0200		Unknown		1.64	
	NP_832874	BC3133		Unknown			−1.86
	NP_833260	BC3527		Unknown		1.75	

*Exoproteins with abundance level restored in ΔmsrAB/pHT304msrAB are indicated in bold. EE, early exponential growth phase; LE, late exponential growth phase; S, stationary growth phase. NA, Not Annotated. Green and red highlights indicate increased and decreased protein levels, respectively*.

Several proteins classified as degradative enzymes showed higher abundance levels in Δ*msrAB* compared with WT (Table 2) and could contribute to the high protease activity of the Δ*msrAB* extracellular milieu (Figure 3B). Interestingly, we showed that the abundance level of Npr600, a predicted bacillolysin, was restored in Δ*msrAB*/pHT304*msrAB* at LE phase. Npr600 could thus be a major contributor to the protease activity of Δ*msrAB* at LE phase (Altincicek et al., 2007).

### *msrAB* regulates the dynamic of the Met(O) content of the *B. cereus* proteome

**Figure 5:**
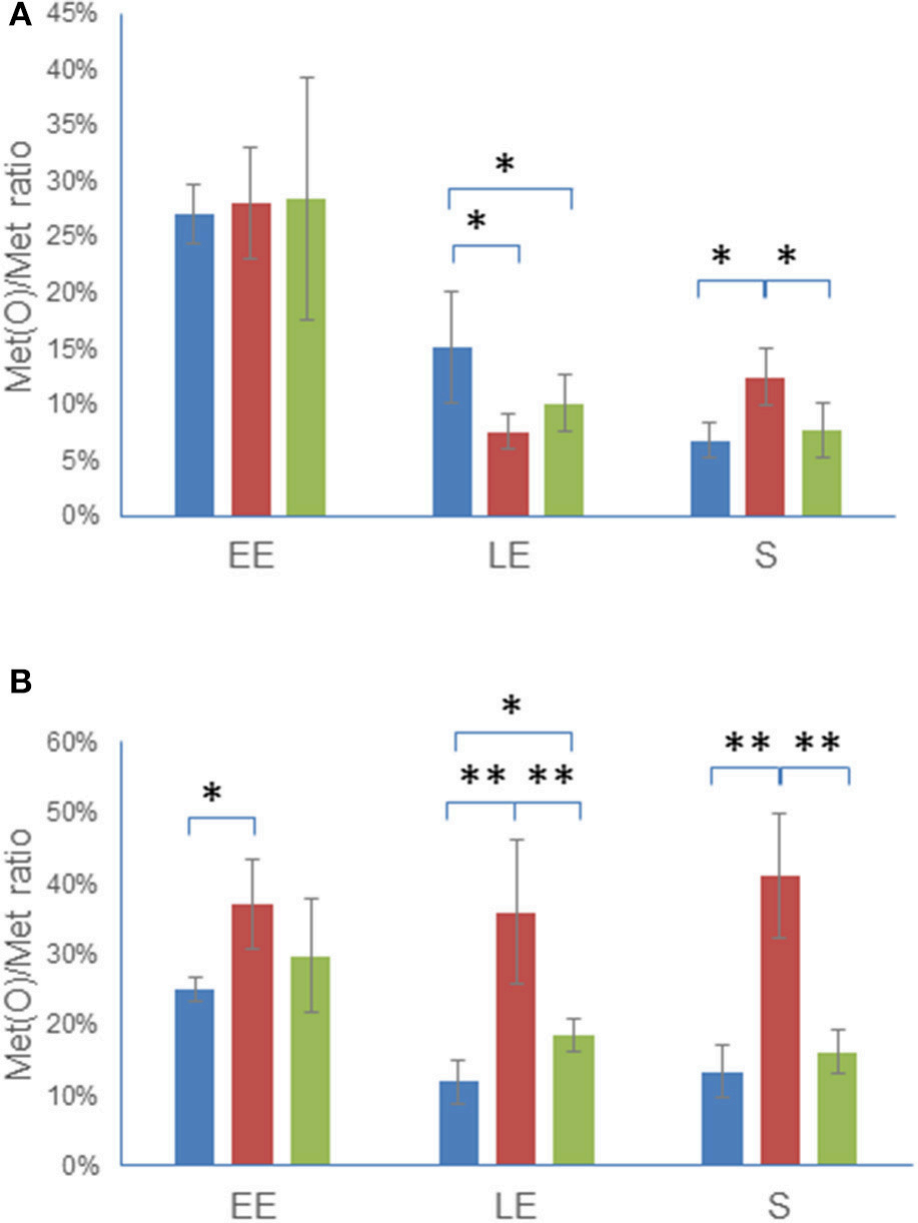
Dynamics of Met(O) content in Δ*msrAB* mutant cells and complemented Δ*msrAB*/pHT304*msrAB* cells. WT (blue), Δ*msrAB* (red), and Δ*msrAB*/pHT304msrAB (green) were grown in MOD medium as described in Figure 2. The cells were harvested at EE, LE and S growth phases. The Met(O) content of the intracellular proteome **(A)** and exoproteome **(B)** was calculated as the percentage of the number of Met(O) vs. the total number of Met residues. Data are the means of triplicate measures obtained from three independent cultures at the EE, LE, and S growth phases. Significant differences between two strains are indicated with one (*p* < 0.05) or two (*p* < 0.01) asterisks.

We identified peptides with oxidized Met in Δ*msrAB*, Δ*msrAB*/pHT304*msrAB*, and WT, in both the cellular proteome and the exoproteome at EE, LE, and S growth phases, as previously described (Madeira et al., 2015). The Met(O) content of both the cellular proteome and the exoproteome was estimated by comparing the number of Met(O) to the total number of Met residues identified in each of the three biological samples obtained for each growth phase in each of the three strains (Tables S4, S5). Figure 5 shows that the Met(O) content of WT and Δ*msrAB*/pHT304*msrAB* decreased similarly in the cellular proteome (Figure 5A) and exoproteome (Figure 5B) during growth. The Met(O) content of the Δ*msrAB* intracellular proteome also decreased during exponential growth and was lower than the Met(O) content of WT at LE phase and higher at S growth phase. More importantly, the Met(O) content of the Δ*msrAB* exoproteome remained constant during growth and accounted for 38 ± 3% of total Met residues. Taken together, these results indicate that MsrAB regulates the dynamic of the Met(O) content of the proteome, especially at the exoproteome level.

To identify peptides exhibiting significant differences in Met(O) content in Δ*msrAB* compared with WT, we conducted a *t*-statistical analysis. For a robust analysis, we considered a peptide as containing an oxidized Met residue when it was identified in at least two biological replicates. The lists of intra- and extracellular peptides showing significant Met(O) level changes (log_2_ fold-change > 1 and *p* ≤ 0.01) according to growth phase are presented in Tables 3, 4.

**Table 3:** Cellular peptides with significant Met(O) level changes (|log_2_|fold-change>1, *p* < 0.01) in Δ*msrAB* compared with WT.

	**Gene no**	**NP no**	**Protein name**	**Description**	**Peptide name**	**Met(O) petide detected by GC-MS/MS**	**log**_**2**_**fold-change**		
							**EE**	**LE**	**S**
Glycolysis	BC3971	NP_833690	PdhC	Alpha-keto acid dehydrogenas,e subunit E2	PdhC_2	HTAPHVTLMDEVDVTELVAHR		−2.32	
Amino sugar and nucleotide sugar metabolism	BC5288	NP_834951	MurA	UDP-N-acetylglucosamine 1-carboxyvinyltransferase	MurA_1	ASVQVMGPLLAR	2.38		
Amino acid metabolism	BC1238	NP_831022	TrpA	Tryptophan synthase, subunit α	TrpA_4	EVQMPFVLMTYLNPVLAFGK		−1.69	
	BC1237	NP_831021	TrpB	Tryptophan synthase, subunit β	TrpB_1	ETPLYYAENMTK		−1.57	
	BC1232	NP_831016	TrpE	Anthranilate synthase component I	TrpE_2	AMEIINELENEKR		−1.02	
Transcriptional regulators	BC0102	NP_829983	ClpC	Negative regulator of genetic competence	ClpC_6	VIELSMDEAR	−3.29		
					ClpC_8	VMTLDMGTVVAGTK	−1.99		
					ClpC_9	VMTLDMGTVVAGTK	−2.40		
	BC0613	NP_830430	ArsR1	ArsR family transcriptional regulator	ArsR1_1	ISEEDVQMLR			1.51
Stress response	BC5044	NP_834714	Dp2	Non-specific DNA-binding protein	Dps2_12	KGMEIAQDSDDEMTSDLLLGIYTELEKHAWMLR			−1.86
		NP_834714			Dps2_9	GMEIAQDSDDEMTSDLLLGIYTELEKHAWMLR			−2.20
	BC0377	NP_830216	AhpC	Alkyl hydroperoxide reductase C22	AhpC_3	IEYIMIGDPTR	−2.34		
	BC0376	NP_830215	AhpF	Alkyl hydroperoxide reductase subunit F	AhpF_18	VSAGDDNVSKDMLALVDELATMSSK			1.14
	BC1155	NP_830941	KatE	Catalase	KatE_5	MNPNNRLTTNQGAPVGDNQNSRTAGR		2.00	
Chaperone	BC0295	NP_830146	GroEL	Chaperone	GroEL_23	SALQNAASVAAMFLTTEAVVADKPEPNAPAMPDMGGMGMGGMGGMM			−1.67
					GroEL_30	SALQNAASVAAMFLTTEAVVADKPEPNAPAMPDMGGMGMGGMGGMM			−1.51
	BC1043	NP_830829	PrsA1	Peptidylprolyl isomerase	PrsA1_6	QVLNNMVMEK		−1.59	
Riboflavin metabolism	BC4112	NP_833830	RibH	Riboflavin synthase, subunit β	**RibH_1**	**AGNKGYESAVAAIEMAHLSK**			2.88
					RibH_3	AGTKAGNKGYESAVAAIEMAHLSK		1.98	2.36
					**RibH_4**	**GVASLSLQMDIPVIFGVLTTETIEQAIER**			2.75
					RibH_5	GYESAVAAIEMAHLSK			2.21
					RibH_8	MASSGKYDAVITLGTVIR			1.93
					RibH_9	MVFEGHLVGTGLK			1.92
	BC4110	NP_833828	RibE	Riboflavin synthase, subunit α	RibE_2	VGSMTESFLQENGFL			1.32
Ribosome components	BC0146	NP_830026	RplF	50S ribosomal protein L6	RplF_1	ALIGNMVEGVTEGFAR			−1.81
	BC3825	NP_833546	RpsB	30S ribosomal protein S2	RpsB_1	AGMYFVNQR		−2.93	
	BC0135	NP_830015	RpsS	SSU ribosomal protein S19P	RpsS_4	KHVPVYITEDMVGHK		2.20	
Translation apparatus	BC0129	NP_830009	Tuf	Elongation factor	Tuf_1	ETDKPFLMPVEDVFSITGR		−1.90	
					Tuf_21	TTDVTGIIQLPEGTEMVMPGDNIEMTIELIAPIAIEEGTK		−1.76	
					Tuf_30	VGDVVEIIGLAEENASTTVTGVEMFRK			1.57
					Tuf_5	IIELMAEVDAYIPTPERETDKPFLMPVEDVFSITGR		−2.95	
Degradative enzyme	BC1991	NP_831760	TgC	Murein endopeptidase	TgC_2	NIMDQLYGEFNK			−1.94
Motility	BC1654	NP_831431	CheV	Chemotaxis protein	CheV_3	VIYIAEDSAMLR	1.75		
Uncategorized	BC1225	NP_831009	–	Unknown	BC1225_1	MKLGIVIFPSK			1.70
	BC4045	NP_833763	–	NAD(P)H nitroreductase	BC4045	MSVEQVSEWAK			1.14
	BC4182	NP_833896	Gls24	Unknown	Gls24_3	AEHMLDMGQDTTLGKVEIAPEVIEVIAGIAAAEVEGVAAMR		−1.02	

*Peptides with Met(O)levels restored in ΔmsrAB/pHT304-msrAB are indicated in bold. Met residues that are differentially oxidized are indicated in red. EE, early exponential growth phase; LE, late exponential growth phase; S, stationary growth phase. NA, Not annotated. Green and red highlights indicate increased and decreased protein levels, respectively*.

**Table 4:** Exopeptides with significant Met(O) level changes (|log_2_|fold-change > 1, *p* < 0.01) in Δ*msrAB* compared with WT.

**Functional class**	**Gene no**	**NP no**	**Protein name**	**Description**	**Peptide name**	**Met(O) petide detected by GC-MS/MS**	**log**_**2**_**fold-change**		
							**EE**	**LE**	**S**
Carbohydrate metabolism	BC5135	NP_834803	Eno	Enolase	Eno_2	LGANAILGVSMAVAHAAADFVGLPLYR	−2.83	−2.67	−2.73
Amino acid metabolism	BC0344	NP_830183	RocA	*1*-pyrroline-5-carboxylate dehydrogenase	RocA_4	FMEVLEEAGLPAGVVNFVPGNGSEVGDYLVDHPR	−2.02		
Translation	BC0119	NP_830000	RplJ	Ribosomal protein L10	**RplJ**	EGLLSMLLSVLQAPIR	−2.21		
Cell wall and cell surface metabolism	BC5234	NP_834897	CwlC	N-acetylmuramoyl-L-alanine amidase	CwlC_1	SGPSHMGIYLGGGSFIQAGDK			2.44
	BC0679	NP_830492	Smc	Cell wall protein	Smc_3	MNAVSTILEADKEILR	2.35		
					Smc_1	GYNLTANPGMK	1.80		
Enterotoxins	BC5239	NP_834902	EntA	Enterotoxin A	**EntA_2**	**VLTAMGHDLTANPNMK**		−1.74	
					**EntA_1**	**VLTAMGHDLTANPNMK**			−2.47
					EntA_3	VLTAMGHDLTANPNMK			1.87
	BC0813	NP_830603	EntC	Enterotoxin C	**EntC_1**	**GNKIDVLMPDK**	2.32		
					**EntC_3**	**IDVLMPDK**		1.65	
	BC3716	NA	EntD	Enterotoxin D	**EntD_1**	**VLTAMGHDLTANPNMK**		1.77	
	BC3102	NP_832845	HblB	HBL, component B	**HblB_6**	**SMNAYSYMLIK**		2.39	
					**HblB_2**	**QLESDGFNVMK**			2.21
	BC3104	NP_832847	HblL2	Hbl, component L1	**HblL2_7**	**LIQTYIDQSLMSPNVQLEEVTALNTNQFLIK**	−3.10	−1.87	−2.18
					**HblL2_9**	**SMLLLTQNDLHTFANQIDVELDLLK**	−2.21		
					**HblL2_10**	**SMLLLTQNDLHTFANQIDVELDLLKR**	−2.49		−1.93
					**HblL2_12**	**TQEYDLMKVIDTEK**		1.96	
					**HblL2_8**	**QDMKEWSSELYPQLILLNSK**			2.09
	BC3523	NP_833256	HlyII	Hemolysin II	HlyII_1	ALEEQMNSINSVNDKLNK	−2.21		
	BC1809	NP_831582	NheA	Nhe component A	**NheA_2**	**LIDLNQEMMR**		2.21	
	BC1810	NP_831583	NheB	Nhe, component B	**NheB_3**	**TQTEYLTNTIDTAITALQNISNQWYTMGSK**	−2.21	−2.27	−1.65
					**NheB_2**	**TGSNALVMDLYALTIIK**		−1.76	
Flagella	BC1657	NP_831434	FlaA	Flagellin	**FlaA_9**	**LDHNLNNVTSQATNMASAASQIEDADMAK**	−2.21	−1.67	
					**FlaA_6**	**ILNEAGISMLSQANQTPQMVSK**		2.92	
					FlaA_5	ILNEAGISMLSQANQTPQMVSK		2.19	
					**FlaA_4**	**ILNEAGISMLSQANQTPQMVSK**		1.78	
					FlaA_20	MRINTNINSMR			−1.78
	BC1658	NP_831435	FlaB	Flagellin	FlaB_7	ILNEAGISMLSQANQTPQMVSK		−2.77	
					FlaB_8	ILNEAGISMLSQANQTPQMVSK		−2.77	
					FlaB_9	ILNEAGISMLSQANQTPQMVSK		−2.77	
					FlaB_14	LDHNLNNVTSQATNMAAAASQIEDADMAKEMSEMTK		−2.29	
					FlaB_11	LDHNLNNVTSQATNMAAAASQIEDADMAK		−2.03	
					FlaB_15	LDHNLNNVTSQATNMAAAASQIEDADMAKEMSEMTK		−1.67	
					FlaB_12	LDHNLNNVTSQATNMAAAASQIEDADMAK		−1.34	
					FlaB_26	TNFNGNSFLDTTATPPGKDIEIQLSDASGDTMTLK		−1.52	−2.06
Degradative enzymes	BC2735	NP_832488.	NprP2	Bacillolysin	NprP2_3	FEAATPNYVSGTYLVNAQNGDMLK		−1.61	
	BC3761	NP_833485	PlcA	1-phosphatidylinositol phosphodiesterase precursor	**PlcA_4**	**WMQPIPDNIPLAR**		2.39	
	BC1991	NP_831760	TgC	Putative murein endopeptidase	**TgC_3**	**NIMDQLYGEFNKIVDADEYVK**		−2.09	
					TgC_10	YKQSMDGTMQDIKK		−2.31	
					**TgC_2**	**NIMDQLYGEFNK**		1.15	
	BC5135	NP_834895	YvgJ2	phosphoglycerol transferase	**YvgJ2_2**	**DIEYFDQSIDMLK**	2.18		
Uncategorized	BC2077	NP_831845	BC2077	ESAT-6-like protein	BC2077	VQNFAQLLQEINMQLNK			−2.47
	BC1894	NP_831667	BC1894	Phage protein	BC1894_1	QDTAAGYQILSFVSDLPGGAISSVVVDLNMPK			−2.18

*Exopeptides with Met(O) levels restored in ΔmsrAB/pHT304msrAB are indicated in bold. Met residues that are differentially oxidized are indicated in red. EE, early exponential growth phase; LE, late exponential growth phase; S, stationary growth phase. NA, Not annotated. Green and red highlights indicate increased and decreased protein levels, respectively*.

#### Cellular proteome

The number of peptides with Met(O) content changes was lower at the EE (6) than the LE (13) and S (19) growth phases. Only one peptide, a RibH-related peptide, showed similar changes in the two growth phases (Table 3). At the EE growth phase, we noted that the subunit E2 of the pyruvate dehydrogenase complex (PdhC), which interconnects glycolysis with acetate metabolism, had one peptide with a decreased Met(O) level in EE phase. This could impact the activity of this enzyme and contribute to the metabolic perturbation observed in Δ*msrAB* at EE phase (Figure 2; Martin et al., 2005). At the LE growth phase, the majority of the identified peptides showed a lower Met(O) content in Δ*msrAB* compared with WT at LE phase. This is consistent with the results presented in Figure 4. At the S growth phase, the majority of the identified peptides (12/18) showed a higher Met(O) level in Δ*msrAB* compared with WT. Among these 12 peptides, 6 are RibH-related peptides. Two of these six peptides had their Met(O) level restored in Δ*msrAB*/pHT304*msrAB* (Table 3). RibH contains four Met residues: all of these were more highly oxidized in Δ*msrAB* than in WT at the S growth phase and two were more highly oxidized in Δ*msrAB* than in Δ*msrAB*/pHT304*msrAB*. RibH is thus a target of MsrAB activity and the major contributor to the difference observed between Δ*msrAB* and WT on the one hand, and Δ*msrAB* and Δ*msrAB*/pHT304*msrAB* on the other, at the S growth phase (Figure 5).

#### Exoproteome

Table 4 shows that peptides with differential Met(O) contents belong to 21 proteins, including eight toxin-related proteins. The LE growth phase sustained the highest number of peptides with increased Met(O) levels (10); the majority of these peptides (9/10) had their Met(O) level restored in Δ*msrAB*/pHT304*msrAB*, indicating a direct impact of MsrAB. Among the proteins with increased oxidation of Met residues were the degradative enzyme, PlcA, the flagellin, FlaA, and the four toxin-related proteins, NheA, HblB, EntC and EntD. Only PlcA and HblB showed increased abundance levels at LE growth phase (Table 2). FlaB was the protein for which we detected the largest number of Met(O) peptides and Met residues with differential oxidation (7 Met residues). All of these residues were less oxidized in Δ*msrAB* compared with WT at LE phase. In addition, we observed that FlaB was less abundant in Δ*msrAB* at LE phase (Table 2). The loss of Met-oxidized peptides could thus be due to degradation of protein copies. This is possibly also the case for HlyII (Tables 2, 4). In S growth phase, the peptides with increased Met(O) content belong to the putative N-acetylmuramoyl-L-alanine amidase CwlC and the toxin-related EntA, HblB, and HblL2. All HblL2-bound Met were not equally susceptible to *msrAB* disruption, as one Met residue was more oxidized at the S growth phase, one was more oxidized at the LE growth phase and two were less oxidized, especially at the EE growth phase. Taken together, the results indicate that MsrAB regulates the dynamic of the Met(O) level of the exoproteome by controlling the Met(O) level of target peptides in a growth phase- and protein-dependent manner. Importantly, our results indicate that virulence factors such enterotoxins, degradative enzymes, and flagella components are MsrAB targets.

## Discussion

Methionine (Met) residues in proteins and their recycling by methionine sulfoxide reductases (Msrs) are part of the antioxidant system produced by aerobic microorganisms. The antioxidant system keeps a steady-state control over ROS production-detoxification (Levine et al., 1996; Kim, 2013). The tight regulation of ROS production and detoxification represents the basis for the maintenance of an appropriate redox homeostasis, which is central for growth.

While Met residues in cellular proteins are well-recognized as antioxidants, the relative importance of Met residues in extracellular proteins has hitherto not been established. In this study, we used next-generation proteomics on wild-type *B. cereus* and an MsrAB mutant to demonstrate that Met residues in exoproteins could be reversibly oxidized to Met(O), probably before their exportation. In addition, we provide the first evidence that *B. cereus* can modulate its capacity and specificity for protein export (secretion) through the growth phase-dependent expression of the methionine sulfoxide reductase-encoding gene, *msrAB*.

As reported for other *msr* genes in several bacteria, *msrAB* expression is lower in exponentially grown *B. cereus* cells than in growth-arrested cells. The low level of *msrAB* expression is probably sufficient to maintain a proper activity of the antioxidant system during exponential growth phase. The increased expression of *msrAB* at the end of growth would serve to minimize the accumulation of oxidative damage on ROS-affected molecules (Dukan and Nystrom, 1999). However, the expression level of *msrAB* in *B. cereus* cells is not by itself sufficient to prevent premature growth arrest under full aerobic conditions as growth can be prolonged by overproducing *msrAB*. In WT cells, premature growth arrest allows the cells to survive for extended time periods, suggesting that MsrAB could be a regulator of normal lifespan of *B. cereus* (Koc et al., 2004).

Considering the primary antioxidant function of MsrAB, variation of other antioxidant proteins was expected in MsrAB-deficient cells as a part of putative compensatory mechanisms or due to altered interactions with MsrAB (Alamuri and Maier, 2006). We observed abundance level changes in antioxidant proteins, mainly at LE phase, due to the lack of protection normally conferred by the high expression of *msrAB*. Neutralizing ROS without quelling its production may prove to be onerous to *B. cereus*. Our results indicate that *B. cereus* reprograms its proteome to both counteract and inhibit the formation of ROS in *msrAB*-deficient cells. This proteome modification leads to novel metabolic networks that allow the alleviation of TCA cycle activity, the main metabolic network that supplies NADH for oxidative phosphorylation. When the machinery involved in oxidative phosphorylation is severely impeded by the ROS challenge, glucose uptake is enhanced to satisfy the ATP need by substrate level phosphorylation. Increased carbon flow also maintains constant levels of glycolytic intermediates as macromolecular precursors and boosts carbon flow through the PPP, which produces large amount of NADPH, a key molecule that is used to drive anabolic processes and provides the reducing power to the antioxidative system. PPP is also required for synthesis of the low-molecular-weight bacillithiol (Richardson et al., 2015).

When *msrAB* is disrupted, *B. cereus* accumulates a higher level of Met(O) exoproteins in the growth medium and a lower level of Met(O) cellular protein at LE phase. This suggests that *B. cereus* can overcome the lack of MsrAB activity by promoting export of Met(O) proteins to maintain intracellular redox homeostasis. Our results indicate that MsrAB deficiency promotes export of some proteins by directly or indirectly modulating the efficiency of the translocation/secretion machinery. Among these proteins are proteases, which probably contribute to the high proteolytic activity of the growth medium of *msrAB*-deficient cells and the highly reduced exoprotein level at the end of growth (Figure 3). Upregulation of proteases has been reported in several bacteria as part of the secretion stress response, which is induced to prevent the accumulation of misfolded proteins outside the cytoplasmic membrane (Westers et al., 2006). MsrAB deficiency leads to the accumulation of oxidized proteins, and oxidation can induce protein misfolding (Tarrago et al., 2012). Thus, MsrAB deficiency may trigger a secretion stress response likely to degrade the misfolded proteins, which could interfere with the correct functionality of the cell (Sarvas et al., 2004). In conclusion, *msrAB* expression may prevent extracellular accumulation of faulty proteins to avoid negative effects in the exported/secreted proteins.

We have shown previously that Met residues in toxin-related proteins may act as ROS scavengers before being secreted (Madeira et al., 2015), and we report here that Met(O) in toxin-related proteins are MsrAB substrates. This indicates that Met residues in toxin-related proteins contribute to the endogenous antioxidant system (Levine et al., 1996, 1999; Luo and Levine, 2009; Kim, 2013), and thus to the cellular redox homeostasis of *B. cereus* (Duport et al., 2016). The reversible oxidation of Met to Met(O) has been suggested to be a mechanism for modulating protein activity (Kanayama et al., 2002). Therefore, catalyzed reduction of Met(O) in toxin-related proteins could be an antioxidant mechanism and a protein regulatory mechanism. This raises important questions about the role of this modification in the biological activity of toxins, and thus in the cytotoxicity of *B. cereus* according to growth phase.

## Author contributions

JM and CD designed the whole experiments. BA and JA helped to design proteomic experiments. JM carried out experiments. CD wrote the manuscript and all authors approved the final manuscript.

### Conflict of interest statement

The authors declare that the research was conducted in the absence of any commercial or financial relationships that could be construed as a potential conflict of interest.
