# Supplementary material for: Methionine Residues in Exoproteins and Their Recycling by Methionine Sulfoxide Reductase AB Serve as an Antioxidant Strategy in Bacillus cereus
Source: Front Microbiol. 2017 Jul 26;8:1342. doi: 10.3389/fmicb.2017.01342 (PMC5526929; doi:10.3389/fmicb.2017.01342)
Supplement: Supplementary file 6 [file DataSheet1.DOCX]

**A.**

TCATGCGCTTGAAAGTTACGTATTAAACCCGAAGTTTATGTCGCAAAAAACAAATTTACC

GATTTTCTATACATTTATGGTATTAATCTTCTCAGAACACTTCTTAGGTGTATGGGGATT

AATTATCGGTATTCCAATCTTCATCTTCTTATTAGATGTTTTAGACGTGACAAGTGATGA

AATGGAGAAGGATGTTGGAAAAAATAAAGTGAAGTAAAGAAAAAGCATCGATGTTCGGTG

CTTTTTCTTTTATAATATGATGTTTCTTATGCCATACTGTAAGCAGAACATAGAATAGGG

GTGAAGTA**ATG**GCTGTAAATGAAAAGGTAGAATTGGCTACATTTGCTGGAGGGTGCTTCT

GGTGTATGGTTTCGCCATTTGAAGAGATGGAAGGGATTATAAAGGTTGTTTCTGGCTACA

CAGGTGGTCATAAAGAGAATCCAACGTATAAAGAAGTATGTTCAGAAACGACGGGACATT

ATGAGGCAGTACAAATTACATTTGATGCAAATAAAATGCCGTATGAGGAATTGTTAAATA

TATATTGGAGACAAATTGATCCGACCGATATTGGTGGACAATTCCACGACCGCGGACAGT

CTTATGAAACGGTGATTTTTTATCATAATGAAGAACAACATAAAAAAGCAGAGGCCTCAA

AAGAAGAGCTTGAGCAGAGTGGACGCTTTTCAAAACCAATTGCGACAAAAATACTACCAG

CAGCTACATTTTATCCAGCTGAAGAATATCACCAAGGATACCATAAGAAAAACACATTCC

GCTATGAGTTATATCGTAAAGGCTCAGGACGAGATGCTTTTATTAAGCAACATTGGCCAA

AGGATAATGCTCATTTAAAAGAAAAACTCAATGAGATGCAGTATTATGTAACACAGGAAA

ATGGTACAGAACCACCATTTCGAAATGAGTATTGGAACCATAAAGAGGAAGGTCTTTATG

TAGACATCGTTTCAGGTGAGCCGTTGTTTACTTCTCTAGATAAATTTGATAGTGGATGTG

GATGGCCTAGTTTTACAAAACCAGTGATGTCAGCAAGTGTGAAAGAAAAGATGGATGTGA

GTCATAATATGACACGTACAGAAGTGAGAAGTAAAGAAGGAGATTCACATCTTGGGCACG

TATTTCCAGATGGTCCAGGACCAAATGGCCTTCGTTACTGTATTAATTCCGCAGCTCTTC

GATTTATTCCAAAAGAGGAATTAGAGAAAGAAGGCTATAGCGATTTCCTAATCTTGTTTG

GAAATAAAAAA**TAA**CCTCGCAGTTAGCGAGGTTATTTTGTTTTTTTCTTTTTAAATTTGC

TTAATACTTCATAAACGATTGGAACAATAAGAAGTGTTAATAACGTTGAACTTGTTAATC

CACCAATTACCGTTACGCCAA

**B.**

mavnekvelatfaggcfwcmvspfeemegiikvvsgytgghkenptykevcsettghyea

vqitfdankmpyeellniywrqidptdiggqfhdrgqsyetvifyhneeqhkkaeaskee

leqsgrfskpiatkilpaatfypaeeyhqgyhkkntfryelyrkgsgrdafikqhwpkdn

ahlkeklnemqyyvtqengteppfrneywnhkeeglyvdivsgeplftsldkfdsgcgwp

sftkpvmsasvkekmdvshnmtrtevrskegdshlghvfpdgpgpnglrycinsaalrfi

pkeelekegysdflilfgnkk

**Figure S1. Genetic organization of *msrAB* and amino acid sequence of its product MsrAB.**

**(A) Nucleotide sequence of the 1401 bp sequence encompassing *msrAB* and cloned into pHT304**. The start and stop codons of *msrAB* are in red and boldfaced. The transcriptional start site is highlighted in yellow. The putative -35 and -10 boxes are in blue. The putative transcriptional terminators of BC_5437(located upstream *msrAB*) and *msrAB* are double underlined.

**(B)** **Amino acid sequence of MsrAB**. Blue peptides were detected both in WT and Δ*msrAB*/pHT304*msrAB* cellular proteome. Red peptides were detected only in Δ*msrAB*/pHT304*msrAB* cellular proteome. Experiments were realized using a Q-exactive HF mass spectrometer.
